# Can the Total Body Water and Total Fat Mass Predict Kidney Stone Recurrence in Overweight and Obese Patients?

**DOI:** 10.7759/cureus.73367

**Published:** 2024-11-10

**Authors:** Catalin Pricop, Marius Ivanuta, Mihaela Nikolic, Ana-Maria Ivanuta, Gina Botnariu, Andreea Elena Stan, Dragos Puia

**Affiliations:** 1 Urology, University of Medicine and Pharmacy "Grigore T. Popa", Iasi, ROU; 2 Urology, "Dr. C. I. Parhon" Clinical Hospital, Iasi, ROU; 3 Center for Morphological and Spectroscopic Analysis of Urinary Stones “Michel Daudon”, "Dr. C. I. Parhon" Clinical Hospital, Iasi, ROU; 4 Nutrition, “Ion Ionescu de la Brad” Iasi University of Life Sciences, Iasi, ROU; 5 Emergency Department, "Sf. Spiridon" County Clinical Emergency Hospital, Iasi, ROU; 6 Diabetes, Nutrition, and Metabolic Diseases, University of Medicine and Pharmacy "Grigore T. Popa", Iasi, ROU; 7 Diabetes, Nutrition, and Metabolic Diseases, "Sf. Spiridon" County Clinical Emergency Hospital, Iasi, ROU; 8 Anesthesiology and Intensive Care, Regional Institute of Oncology, Iasi, ROU

**Keywords:** obesity, prevention of kidney stones, total body water, urolithiasis, water deficit

## Abstract

Background

Urolithiasis prophylaxis is a cornerstone for kidney stone formers. Obesity is a well-known risk factor for kidney stone recurrence. The objectives of this research were to highlight the correlations between the mean water intake and free water deficit (FWD) depending on plasma Na and weight.

Methods

Anthropometric and nutritional analysis was performed using a body analyzer with magnetic bioimpedance ankle-to-foot, which determined the total percentage of water in the body.

Results

The mean age was 52.19 years old and the mean body mass index (BMI) was 33.68 km/m^2^. About 58.23% (n=46) of patients declared at least one episode of urolithiasis in their pathological antecedents. The patients were counseled by a dietician and lost weight. After losing weight, the total body water (TBW) percentage increased (average=41.37%), while the total fat percentage decreased (average=33.52%). Urinary volume increased, but the water did not accumulate for proper hydration. This may explain the recurrence of urinary stones despite large amounts of water intake.

Conclusions

The present study indicates an inverse-proportional relationship between TBW and fat mass (FM). Obese patients with lithiasis should lose weight due to total FM, not TBW.

## Introduction

Nephrolithiasis is a common condition that can be particularly prevalent in obese patients. The pathophysiology of nephrolithiasis in obese patients involves several complex mechanisms, including metabolic, dietary, and anatomical factors. Nephrolithiasis in obese patients is primarily influenced by insulin resistance, nutritional factors, and a lithogenic urinary profile, leading to uric acid stones and calcium oxalate stones. The pathophysiology involves altered renal acid-base metabolism due to insulin resistance, resulting in a lower urine pH and an increased risk of uric acid stone disease. Additionally, obesity is associated with excessive intake of lithogenic substances and a higher incidence of urinary tract infections, further contributing to stone formation. Bariatric surgery, increasingly utilized for obesity management, can exacerbate nephrolithiasis risk by causing fat malabsorption, leading to hyperoxaluria and other metabolic derangements in urine composition [1]. Understanding these mechanisms is crucial for implementing preventive measures such as dietary modifications, fluid intake optimization, and appropriate supplementation to mitigate the risk of kidney stone formation in obese individuals.

Hydration is crucial in developing nephrolithiasis by influencing urine volume and composition. Studies emphasize the importance of increasing fluid intake to prevent kidney stone formation and recurrence [2]. Research indicates that encouraging a daily water intake of over 2500 mL and maintaining a urine output of 2 L/day can lower the prevalence of nephrolithiasis [3]. In pediatric cases, water consumption of around 2 to 3 L/m^2^ per day is recommended, with specific genetic conditions requiring even higher hydration levels [2]. Furthermore, pediatric nephrolithiasis trends have been linked to fluid intake, nutrition, and lifestyle changes, highlighting the need for further research. Overall, adequate hydration, balanced fluid intake, and urine dilution are essential strategies for preventing and managing nephrolithiasis.

Water is the principal compound in the human organism and an essential regulator in its internal environment. Mean values for total body water (TBW) are approximately 38 to 46 L in white men and about 26 to 33 L in white women. Intracellular water (ICW) is approximately 65% of TBW and 35% is extracellular in a 70 kg person. Numerous diseases, particularly renal insufficiency, affect TBW. Therefore, predicting TBW volume in renal disease is critical to prescribing and monitoring treatment [4,5].

Clinicians and researchers have reported that individuals of the same age, height, and weight can have different body shapes, body composition, energy requirements, and metabolic profiles [6]. The accuracy of TBW measurement is demanding, requiring isotopic dilution techniques that are not readily applicable to the clinical setting. Researchers and clinicians commonly use indirect methods to estimate TBW [7]. There is no perfect method. Some of them are not without consequences. CT scan or dual-energy X-ray absorptiometry (DEXA) uses X-rays and cannot be applied in some individuals (children and pregnant women). In contrast, other methods are considered less precise. Our goal was to establish correlations among the mean water intake reported by obese lithiasic patients, free water deficit (FWD) based on plasma Na levels, and weight, given that many obese patients have the misconception that reducing water intake helps with weight loss.

## Materials and methods

From February 2021, we initiate a complex ongoing program for evaluation and monitoring weight loss and kidney stone recurrence in obese lithiasis patients. The study takes place in the Urology Department of the Clinical Hospital "Dr. C.I.Parhon", in collaboration with the Diabetes, Nutrition, and Metabolic Diseases Department within the Emergency Clinical Hospital" St. Spiridon", from Iasi. A dietitian assists with the recommendations for weight loss, preventing lithiasis recurrence is a long-term goal. This study was approved by the Research Ethics Committee of "Dr. C.I.Parhon" Hospital (7618/26.09.2022)

The inclusion criteria in this study were a documented history of kidney stones and a body mass index (BMI) corresponding to overweight or obesity. Patients who did not agree to participate in this study, patients with concurrent neoplastic pathologies, and transplanted patients were excluded from the study.

In this study, all participants completed a 40-item food frequency questionnaire developed in our clinic. The questionnaire was specifically tailored to investigate dietary habits, associated pathologies, and other relevant factors in the context of renal lithiasis. The questionnaire was designed to comprehensively assess lifestyle elements, including eating habits, fluid intake, current treatments for chronic diseases, and physical activity levels, to better understand the potential risk factors and health behaviors that may influence the development and recurrence of kidney stones. Each participant underwent clinical tests following the questionnaire to collect baseline health information. These included a complete blood count to screen for overall health and potential infections, an ionogram to assess electrolyte balance, and a C-reactive protein (CRP) test to measure systemic inflammation. Additional tests were conducted to evaluate renal and liver function, total cholesterol, triglycerides, and uric acid levels, all relevant to metabolic health and the risk of lithiasis. Finally, a comprehensive urinalysis was performed to detect urinary anomalies related to kidney stone formation.

After these assessments, patients were referred to a dietitian for an in-depth anthropometric and nutritional evaluation. This was performed using a body composition analyzer based on magnetic bioimpedance technology (Beurer model BF 1000, ankle-to-foot). This device measured each patient's weight, abdominal circumference, and body water percentage, providing essential data on body composition, hydration status, and potential risk factors linked to obesity and urolithiasis.

FWD was calculated based on total body weight (TBW) and plasma Na with the formulas: for men: FWD = 0.6 x TBW (kg) x (Measured Na/Ideal Na-1) and for women: FWD = 0.5 x TBW (kg) x (Measured Na/Ideal Na-1).

Although sodium's usual physiological range is considered to be between 135 mmol/L and 145 mmol/L, according to Cheuvront et al., a sodium level of 140 mmol/L plays a crucial role in maintaining physiological homeostasis, impacting blood pressure regulation, nervous system signaling, and extracellular fluid volume control in the human body [8]. For this reason, the ideal Na value was considered 140 mmol/L.

Statistical analyses were performed with Microsoft Excel (Microsoft Corporation, Redmond, Washington, USA) and SPSS Version 26 (IBM Corp., Armonk, NY, USA). We applied tests for descriptive statistics, and the correlation between TBW, fat mass (FM), and FWD was determined with the Excel function "Correlation". The interpretation is according to the Pearson coefficient. We also performed ROC curve analyses with SPSS.

## Results

The present study included 79 patients (42 females and 37 males), which corresponded with all the inclusion criteria. The patients were between 19 and 74 years old, with a mean age of 52.19 (+/-11.8). The mean BMI was between 26.83 and 44.85; the mean BMI was 33.68 km/m^2^, as shown in Table 1.

**Table 1:** Descriptive statistics of the population BMI: body mass index

Parameter	Mean	SD
Age (years)	52.19	11.80
Height (m)	1.68	0.09
BMI (kg/m^2^)	33.68	4.49
Waist circumference (cm)	115.67	10.26
Daily water intake (L/24h)	1.86	0.83
Urea (mg/dl)	34.68	13.18
Creatinine (mg/dL)	0.89	0.25
Urinary pH	5.68	0.64
Na plasma (mEq/L)	140.78	2.08

The mean water intake declared at first evaluation was 1.86 L/day, 22.78% of patients claimed to consume alcohol regularly, 36.71% admitted consuming soda beverages, and 81.01 % drank coffee daily, as shown in Table 2.

**Table 2:** Eating behavior and urologic status

	Alcohol consumption	Sodas	Juice	Coffee	Tea	Urolithiasis recurrence	Urologic interventions
Yes	22.78% (n=18)	34.18% (n=27)	36.71% (n=29)	81.01% (n=64)	54.43% (n=43)	58.23% (n=46)	64.56% (n=51)
No	77.22% (n=61)	68.35% (n=54)	63.29% (n=50)	18.99% (n=15)	45.57% (n=36)	41.77% (n=33)	35.44% (n=28)

Kidney stone recurrence is one of our main preoccupations; at this point, 58.23% (n=46) of patients declared a minimum of one episode of urolithiasis in their pathologic antecedents. The stone composition was assessed through infrared spectroscopy. In patients with a single episode of kidney stones, 76.08% (n=35) had calcium oxalate as the main component, 17.83% (n=8) had uric acid, and 6.52% had carbapatite or brushite. In patients with kidney stone relapse, the last known composition was calcium oxalate (72.72%), uric acid (21.21%), and carbapatite or brushite in 6.07%. For those with a single episode, the mean daily water intake declared was 1.9 L, nine of them admitted to drinking alcohol regularly, 13 (28,26%) drink sodas often, the mean BMI was 33.67 kg/m^2^, and the mean age was 49.58 years old.

The mean TBW for patients with urolithiasis recurrence was 36.71% and FM was 37.4 %. The mean plasma Na was 140.86 mmol/L and the mean urinary pH was 5.73.

We calculated the FWD depending on plasma Na and weight. The mean FWD was 0.26 L for all our patients, but it was 0.33 L for those with kidney stone recurrence. The maximum FWD was 2.21 L for a patient with 27% TBW, 49.5% FM and a BMI of 41.18 kg/m^2^.

With the Excel function Correlation, we analyze the link between TBW, FM, and FWD (Table 3). There is a very weak correlation between FWD and TBW and between FWD and FM, but there is a strong negative correlation between TBW and FM. In other words, when FM is high, TBW is low.

**Table 3:** Correlation between TBW and total FM TBW: total body water; FM: fat mass; FWD: free water deficit

FWD	TBW
1	
-0.10086754	1
TBW	FM
1	
-0.75650319	1
FWD	FM
1	
0.023433706	1

A dietician counseled the patients. The average kilograms lost was 4.05 kg, about 0.6 kg in two weeks. After these changes, the TBW percentage increased, average=41.37%, and the total fat percentage decreased, average=33.52%. The correlation coefficient is closer to -1, indicating a negative correlation between TBW and total FM.

Our study found that the mean water intake declared for obese lithiasis patients was approximately 2 L/day, but the TBW was low. This observation concluded that although they drink enough water, obese lithiasic patients have a degree of dehydration. Nevertheless, after the diet's establishment, they lost weight and increased the total amount of water in their bodies.

Another important aspect is the regulation of urinary pH. Changing the urinary pH by urine alkalinization at the beginning and later by adapting the diet reduces the risk of lithiasis recurrence. Patients report their urinary pH values to the team daily, both in the morning and evening, and the dietitian changes the menu of patients who declare an abnormal urinary pH value in real time.

The patients were reevaluated periodically, and it is important to mention that, during the bioelectrical impedance analysis, as patients lost weight, FM decreased and the TBW percentage increased.

**Figure 1:**
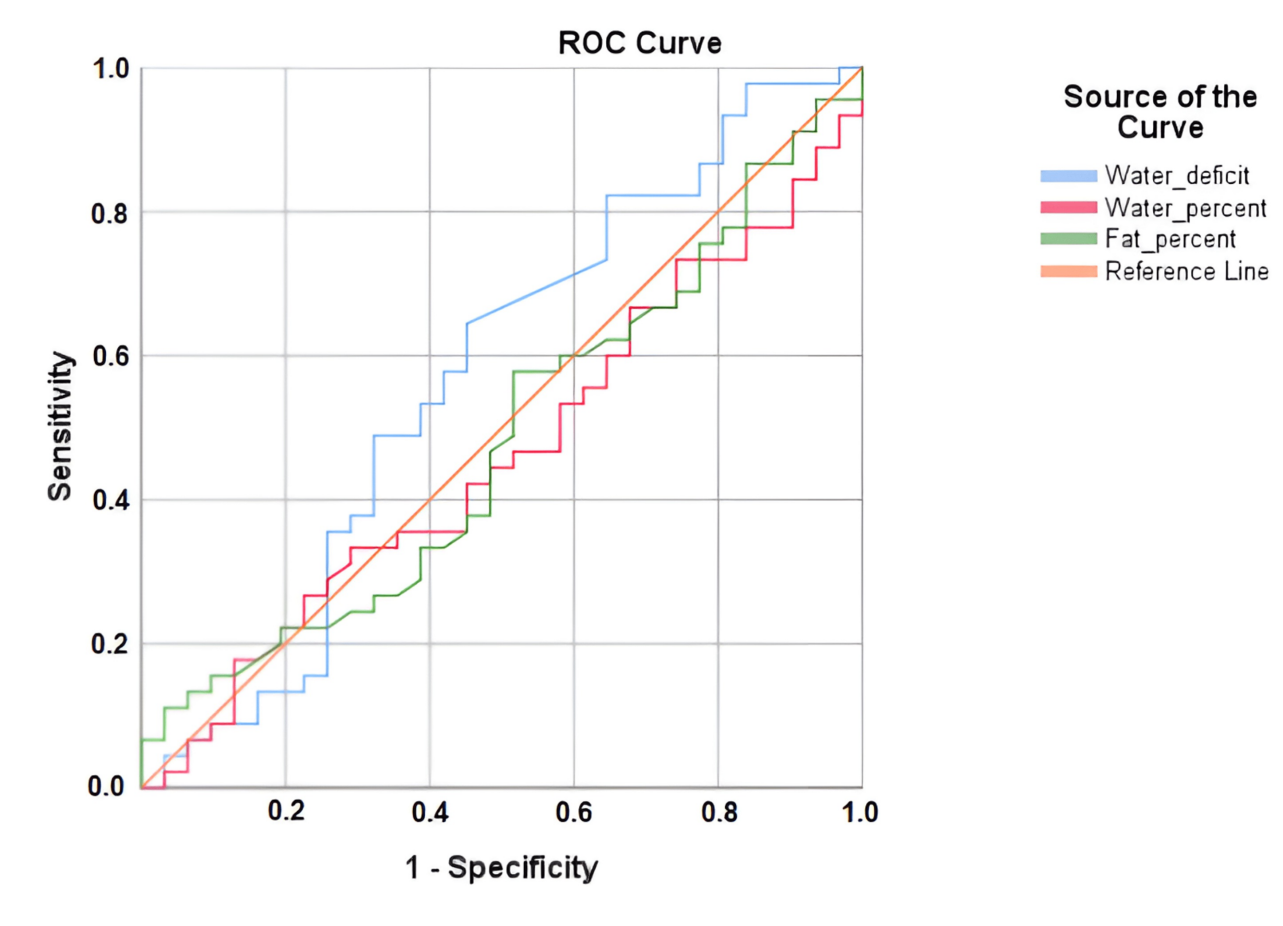
ROC curve analysis showed that total water deficit could predict the recurrence of kidney stones

We performed an ROC curve analysis for total water deficit, TBW percentage, and FM measured with bioimpedance, using the presence of lithiasis as the reference. We found that the total water deficit could predict the recurrence of kidney stones (Figure 1). 

A number of positive outcomes is higher than negative outcomes (N=45), so the probability that FWD predicts a positive outcome for urolithiasis is reflected by the area under the curve: 0.57 (std. error=0.7) for total water deficit, 0.462 (std. error=0.67) for water percentage measured with magnetic bioimpedance, and 0.481 (std. error=0.68) for total fat percentage measured with magnetic bioimpedance.

## Discussion

High liquid intake is the main recommendation for patients with kidney stones, regardless of body weight or stone composition. However, hydration status is influenced by many factors. A higher environmental temperature or a diet rich in foods with low water content and possibly high sodium content can induce a state of dehydration and, therefore, urine with an increased osmolarity. In addition, data in the literature suggest that there is an inversely proportional relationship between the degree of hydration and BMI. Moreover, research conducted by Carretero-Gómez et al. on overweight persons showed a notable correlation between obesity and hydration level, as seen by elevated plasma osmolarity, urine osmolarity, and urinary specific gravity in individuals with higher weight categories [8,9]. In general, obesity has a detrimental effect on hydration status. This highlights the significance of consuming enough fluids in individuals with obesity to ensure correct hydration levels and perhaps avoid related health concerns.

In real life, it is very difficult to assess the correct hydration status of individuals. For many years, the International Atomic Energy Agency (IAEA) has fostered the more widespread use of the stable isotope technique to assess body composition in different population groups. An international group of experts developed this technique to provide analysis of stable isotope ratios in biological samples made by isotope ratio mass spectrometry (IRMS) [10]. TBW can be estimated using the deuterium oxide dilution technique. Deuterium is a stable isotope of hydrogen with the symbol 2H. It is given orally as deuterium oxide (H_2_O_2_), and after mixing with the body, water is eliminated through urine, saliva, sweat, and human milk. The equilibration of deuterium in body water is faster with saliva than with urine [10].

A study by Simpson et al. estimates the comparability of body water compartments in healthy volunteers using electrical impedance analysis with established tritium and NaBr dilution reference methods. They concluded that measuring TBW (tritium or deuterium dilution) and extracellular water (ECW) (sodium bromide (NaBr) dilution) are invasive, expensive, labor intensive, and time-consuming. Also, it cannot be applied repeatedly to monitor patients during therapy. However, dual-frequency BIA provided a reasonably accurate estimate of ECW [11].

Miller et al. published an article from 1999 used the measurement of body composition and TBW in patients with burns to assess hydration, as measured by BIA, and compared it with tritiated water. They find a significant relationship between BIA and tritiated water methods of determining the TBW of patients with severe burns [12].

Bioimpedance analysis is a broadly applied approach used in body composition measurements and healthcare assessment systems [13]. Since the late 18th century, research on the electrical characteristics of biological tissues has been conducted [14]. Bioimpedance is biological tissue's ability to impede electric current. Bioimpedance is a complex quantity composed of resistance (R). It is caused by TBW and reactance (Xc) caused by the cell membrane's capacitance [15,16].

Body composition estimation using bioimpedance measurements is based on determining body volume through the primary means of resistance measurement. The human body is generally composed of FM, which is considered a non-conductor of electric charge. FM equals the difference between body weight (WtBody) and fat-free mass (FFM), as shown in the equation: FM=Wtbody-FFM. FFM is considered the conducting volume that helps pass electric current due to the conductivity of electrolytes dissolved in body water. Studies show that water, known as TBW, is the primary compound of FFM and is equal to 73.2% in normal hydration subjects, as in the equation TBW=0.73xFFM [17].

Measurement of total body bioimpedance is the most commonly used method for estimating whole-body compartments. Many whole-body bioimpedance instruments apply three approaches for impedance measurement: the hand-to-foot method, foot-to-foot, and hand-to-hand method [13].

Segmental bioimpedance analysis achieves better skeletal muscle mass estimation (SMM) than whole-body analysis, with a reported standard error of 6.1% in MRI measurements among 30 male subjects [18]. The body's composition varies among ethnic and racial groups, as well as between men and women [19,20].

In 2005, Jaffrin et al. compared the accuracy of a foot-to-foot impedance meter with a multifrequency bioimpedance using DEXA as a reference. They found that foot-to-foot measurement overestimated FFM by an average of 3% in men and underestimated it by 0.5% in women, but the sums were identical for FFM and FM. Regarding ECW, the values for foot-to-foot, ankle-to-foot, and those measured by electrodes were in good agreement [21]. It is essential to mention that TBW is the sum of ECW and ICW volumes. The ICW resistance cannot be measured directly with bioimpedance but must be calculated. ECW and ICW behave as parallel resistances [22]. It is known that urinary stones occur more often due to nutritional habits, obesity, and a sedentary lifestyle besides endocrine and metabolic causes [23].

The mechanism for increasing water intake is to dilute the lithogenic substances in the urine, and as a consequence, it reduces the risk of developing kidney stones [24]. For each 200 mL of fluids consumed daily, a 13% reduction in the risk of forming urolithiasis was found. Many studies compare bioelectrical impedance measurements of TBW with anthropometric equations, the deuterium dilution method, sodium bromide dilution techniques, or the tritiated water method [25]. 

Preventing nephrolithiasis is the primary concern for kidney stone formers. Therefore, adequate hydration is a principal recommendation to maintain urinary volume over 2 L/day. The mechanism for increasing water intake is to dilute the pro-lithogenic substances in the urine, which reduces the risk of developing kidney stones. For each 200 mL of fluids consumed daily, a 13% reduction in the risk of forming urolithiasis was found according to Littlejohns [25].

High fluid intake is a low-risk, inexpensive, and effective method to prevent urolithiasis. An oral fluid intake is sufficient to cause urine output of at least 2-2.5 L/day [26,27]. However, this recommendation may cause specific challenges to patients with low urinary tract syndrome or overactive bladder because of restricted access to fluids for this category or those with limited access to the bathroom [28,29].

According to Littlejohns et al., fluid intake and dietary factors are associated with the risk of developing kidney stones. They stated that a high fluid intake of fruits and fiber was associated with a lower risk of hospitalization. Modifiable dietary factors could also be targeted to prevent kidney stone development [25]. In 2021, Scales Jr et al. described the Prevention Of Urinary Stones with Hydration (PUSH) study. They conducted a randomized clinical trial that used a ”smart” water bottle to track fluid intake. Their ongoing study is expected to support the role of increased fluid intake in stone recurrence [30]. Similar to this was a study in 2020, where participants with a history of kidney stones used digital tools to track fluid consumption to trigger reminders to drink. After one month's follow-up, the fluid intake increased significantly and remained elevated after three months [31]. These data are confirmed by our previous experience. More than 80% of lithiasis patients referred to our center are overweight or obese. Of these, almost 60% reported reduced consumption (less than 2 L/day) [32].

Another study conducted in 2020 by Joshua Bernard et al. measured the daily water intake of 25 adolescents with nephrolithiasis over seven days. They also measured urine volume over 24 hours within a 12-month period to estimate the "fluid prescription." The median daily water intake was 1.4 L, and the median 24-hour urine volume was 2.01 L. They also stated that a 1 L increase in median daily water intake was associated with a 710 mL increase in 24-hour urine volume [33].

A question that arises: "Does water quality have an impact on the recurrence of kidney stones despite the recommended quantity?" Limited studies have examined the relationship between water quality and urolithiasis. It is stated that prolonged consumption of high-salted water may cause kidney stone formation [34]. There is controversy about how water hardness impacts the development of urinary stones. No significant correlation is found in the literature between water hardness and the incidence of kidney stones [35-38]. However, a study conducted by Bellizzi et al. reported that intake of hard water increases the chance of stone formation by a 50% increase in urinary calcium concentration [39]. This statement was also supported by Coen et al.; they concluded that an increase in drinking hard water increased urinary stone incidence [40]. Mitra et al. stated that water quality is not essential, but quantity matters most in developing kidney stones [41]. They analyzed pH, alkalinity, hardness, total dissolved solutes, electrical conductivity, and salinity from 1266 patients with kidney stones from different areas in West Bengal, India. The water parameters do not differ between the case and control areas [42]. 

Obesity is a disease with long-term effects that affects many people around the globe. However, drinking water is recommended to maintain a healthy hydration status [42,43]. A literature survey related that drinking water is strongly linked as a dietary means for weight loss and overweight/obesity prevention [44]. Fulgoni et al. stated that obese adults consume more plain water than normal-weight adults [45]. Other studies did not support this assertion; Kuczmarski et al. found that water consumption was higher among overweight and obese African Americans than white counterparts, but there was no difference between the normal-weight, overweight, and obese adults [46].

Increased water consumption before each of the three daily meals and a hypocaloric diet showed a reduction in body weight directly correlated with increased water quantity [47]. A similar study stated that premeal water consumption, in addition to a behavioral program for self-monitoring and monthly dietary counseling, resulted in significantly greater weight loss [48].

Stookey et al. published an article in 2008 about dietary programs. They concluded that women who increased their total water consumption over 1 L/day had, on average, a more significant weight loss of 2.3 kg compared with women who drank less than 1 L/day [49]. Similar results were obtained for waist circumference and total body FM.

A prospective study conducted by Paz-Graniel et al. analyzed the PREDIMED-Plus cohort to investigate if there is a link between adiposity changes and water consumption instead of soups, beers, spirits, and hot beverages [50]. They found that elderly Mediterranean participants with high cardiovascular risk who drank large quantities of water showed significant body weight and waist circumference reduction after one and two years of follow-up [51].

An important topic is water intake for patients who have had bariatric procedures, like laparoscopic sleeve gastrectomy or gastric bypass Roux-en-Y. Bariatric surgery causes reduced food tolerance, nutritional deficiencies, and a negative influence on life quality [52]. Therefore, oral liquid tolerance, particularly for water, is essential before hospital discharge.

Athar Elward et al. published a study in 2019 in Obesity Surgery magazine that determined the incidence of reduced water tolerance after laparoscopic sleeve gastrectomy compared with juice and other liquids and assessed weight loss and food tolerance score. They found that 49% of patients experienced early difficulty drinking water, and 29.41% had late difficulty drinking water. Also, the mean food tolerance score was not significantly different between patients with late difficulty drinking water [51]. Other studies also support this statement, as patients with bariatric surgery have a slow gastric passage for food and liquids, thus losing weight [53-57].

Our study has several limitations. Although obesity is frequently found in lithiasis patients, our group was relatively small due to the lack of compliance of many. The TBW determination method does not have a very high accuracy. DEXA is more accurate; it is also more expensive and less accessible. Another limitation is the heterogeneity of the obese population. Obesity is a heterogeneous condition with different fat distributions (e.g., visceral versus subcutaneous fat). This variability can influence TBW differently, making it difficult to generalize findings.

## Conclusions

This study underscores the importance of integrating dietary management with specific treatments for urinary stones, with particular emphasis on weight reduction and balanced nutrient intake. Our findings reveal a significant inverse relationship between TBW and FM, highlighting that simply increasing water intake is insufficient to raise TBW in the absence of weight loss. As fat tissue occupies space that would otherwise contain water, reducing FM is essential for improving hydration status in obese lithiasis patients.

Additionally, the study suggests that a deficit in TBW may predict the recurrence of kidney stones, potentially explaining why high fluid intake alone does not prevent stone formation. These findings support the recommendation for targeted weight loss from FM to reduce lithiasis risk, though further studies are needed to confirm this association and explore underlying mechanisms.
